# Symptoms of anxiety and depression associated with steroid efficacy and clinical outcomes in patients with inflammatory bowel disease

**DOI:** 10.3389/fpsyt.2023.1029467

**Published:** 2023-07-21

**Authors:** Shihao Duan, Yi Yang, Yubin Cao, Pingrun Chen, Chang Liang, Yan Zhang

**Affiliations:** Department of Gastroenterology, West China Hospital, Sichuan University, Chengdu, Sichuan, China

**Keywords:** inflammatory bowel disease, depression, anxiety, steroid resistance, HADS

## Abstract

**Background:**

Anxiety and depression symptoms are very common in patients with inflammatory bowel disease (IBD). We aimed to explore the impact of anxiety and depression on the efficacy of medications, as well as IBD-related poor outcomes.

**Method:**

This was a prospective longitudinal observational study. Hospital Anxiety and Depression Scale was used to assess anxiety and depression symptoms. Logistic regression analyses were used to assess the association between anxiety/depression and the response to different medications. Kaplan–Meier survival analysis and Cox regression model were applied to analyze the relationship between anxiety/depression and IBD-related poor outcomes, which were defined as urgent IBD-related hospitalization, IBD-related surgery, or death.

**Results:**

A total of 325 IBD patients were enrolled, 118 of whom were treated with corticosteroids, 88 with azathioprine/6-mercaptopurine (AZA/6-MP), and 147 with anti-TNF agents. Anxiety/depression symptoms were found to be significantly related to steroid resistance, but independent of AZA/6-MP and anti-TNF agents nonresponse. There was a significant association between anxiety/depression symptoms and IBD-related poor outcomes. Coexisting with anxiety/depression symptoms was an independent influencing factor of steroid resistance and IBD-related poor outcomes.

**Conclusion:**

IBD patients with anxiety/depression symptoms were at a higher risk of developing steroid resistance and IBD-related poor outcomes. Future studies are needed to explore whether interventions for anxiety and depression will improve their response to medications and change their prognosis.

## 1. Introduction

Inflammatory bowel disease (IBD), which includes Crohn’s disease (CD) and ulcerative colitis (UC), is a chronic inflammatory disease of the gastrointestinal tract characterized by episodes of relapse and remission. A growing number of studies have suggested that IBD is highly associated with psychiatric disorders like anxiety and depression (1–5). Patients with a history of depression were more likely to be diagnosed with IBD, and certain antidepressants could protect against IBD (1). The prevalence of depression is reported to be 22–25% and anxiety is reported to be 32–35% in patients with IBD (3, 4). Compared to the general population, patients with IBD have two times the risk of developing anxiety and depression (5). Patients with active disease also had an increased prevalence of depression and anxiety compared with those in remission (3, 6). Besides, IBD patients with anxiety and/or depression are more likely to need treatment and medical resources, and are more challenging to manage (7).

The increased incidence of anxiety and depression in IBD raises the possibility that this condition has an impact on the disease progresses. Some demographic and clinical factors are reported linked to a worse outcome in IBD. For instance, smoking, being diagnosed at a young age, and having perianal disease are risk factors for more severe disease and recurrence in CD (8, 9); younger age and basal plasmacytosis in histological biopsies are associated with relapse in UC (10, 11). But more recently, a growing number of studies have shown that anxiety or depression may increase the risk of poor disease outcomes such as relapse, hospitalization, and intestinal surgery (12–15). A newly published meta-analysis of 9,192 patients also revealed that anxiety and depression were linked to a significantly increased probability of escalation of therapy and emergency department attendance (16). However, a large retrospective study did not discover a connection between them (17).

The complex interaction between psychological disorders and IBD may involve neuroendocrine pathways, including the hypothalamic–pituitary–adrenal (HPA) axis and the central, peripheral and autonomic nervous systems (18). Psychogenic stressors can induce HPA axis activation and neurotransmitter changes in the brain, and ultimately promote the development of glucocorticoid resistance through impaired glucocorticoid receptor (GR) function (19). Also, symptoms of anxiety and depression among individuals without a psychiatric diagnosis are associated with abnormal cortisol responses (20). Even though several molecules have reportedly been linked to glucocorticoid resistance in IBD, the findings are still inconsistent (21). Yet, it is unknown if anxiety and depression would promote glucocorticoid resistance in IBD.

Corticosteroids are the first-line agents for the induction of remission in IBD patients with moderate to severe activity (22). Other important therapeutic agents include immunosuppressants and biologics. However, the association between symptoms of anxiety and depression and the medication efficacy, particularly corticosteroids, have not been reported in IBD. Additionally, their influence on the disease prognosis needs to be further investigated. Therefore, we conducted a prospective cohort study to explore these issues.

## 2. Methods

### 2.1. Patients and study design

This is a prospective longitudinal observational study including patients in West China Hospital of Sichuan University, the largest IBD center in Southwest China, between January 2020 and November 2021. The inclusion criteria were as follows: (1) patients were diagnosed with IBD according to the third European Evidence-based Consensus on Diagnosis and Management of Crohn’s disease and Ulcerative Colitis (23, 24); (2) patients who were willing to participate in our study and can understand and complete the Hospital Anxiety and Depression Scale (HADS) questionnaire; (3) age ≥ 18 years. Patients who met any of the following criteria were excluded: (1) concomitant with other psychiatric disorders (e.g., bipolar disorders and schizophrenia) other than anxiety or depression disorders. (2) patients with severe chronic diseases (e.g., chronic heart failure and chronic obstructive pulmonary disease), other immune disorders or cancer.

**Figure 1:**
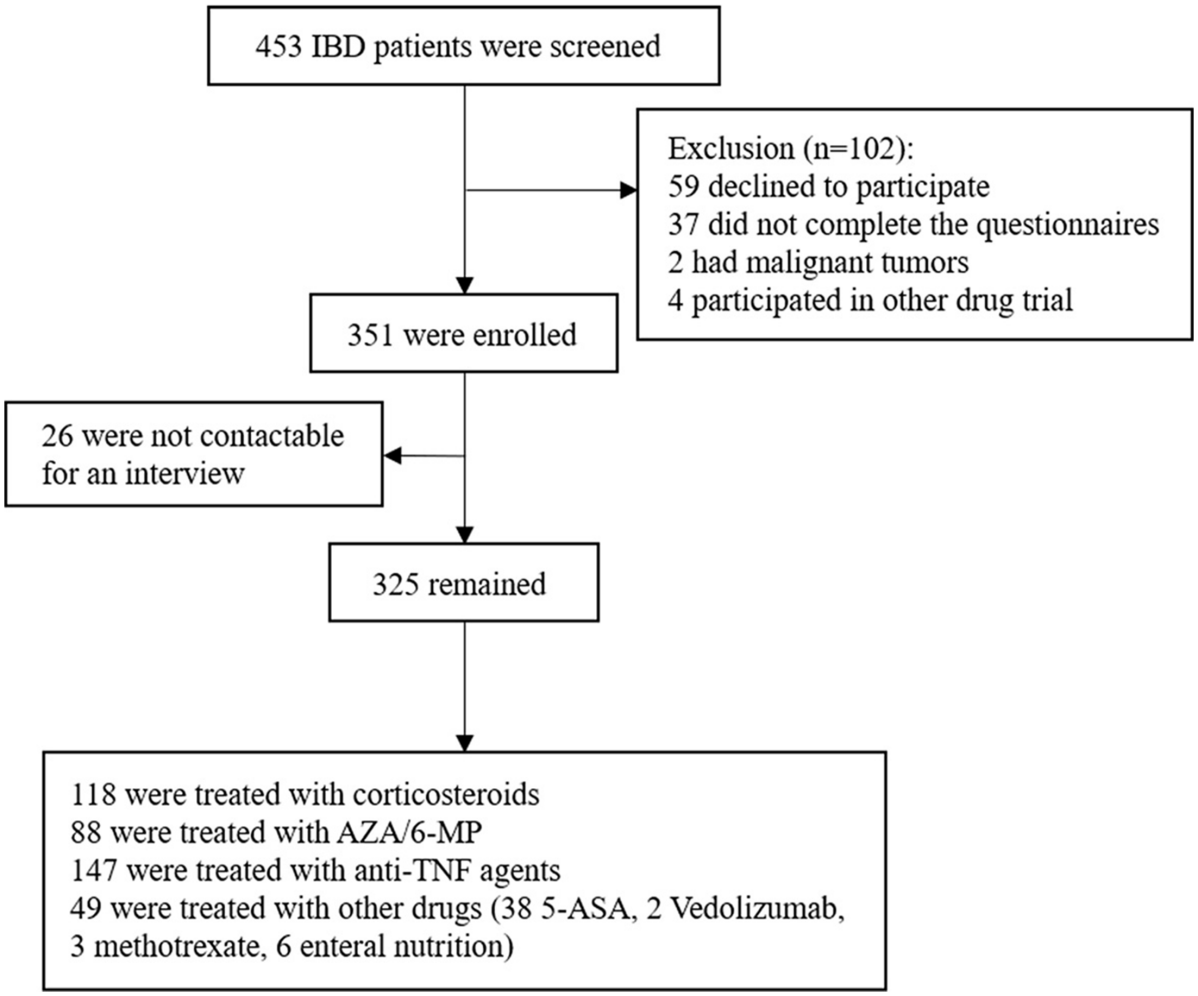
Flow chart for the study participants’ enrollment.

At enrolment, patients were asked to complete the HADS questionnaire at the time of the first clinical encounter recorded within this study period. Laboratory tests and endoscopy would also be completed in the next few days to assess the severity of the disease and microbial infection. Baseline characteristics were collected at the time of enrolment and before treatment begins, including current age, gender, BMI, tobacco use, disease type (CD and UC), previous surgery and other clinical features, and laboratory studies. Patients with anxiety and/or depression symptoms were defined as HADS-anxiety (HADS-A) and/or HADS-depression (HADS-D) score ≥ 8, while both HADS-A and HADS-D score < 8 were defined as without anxiety or depression symptoms. The severity of disease was assessed using the Mayo Clinic score for UC (25) and CD activity index (CDAI) for CD (26). Clinical remission was defined as Mayo <3 or CDAI <150, and clinical active was defined as Mayo ≥3 or CDAI ≥150. The endoscopic score was evaluated by the Simple Endoscopic Score for Crohn’s disease (SES-CD) and Ulcerative Colitis Endoscopic Index of Severity (UCEIS) respectively. Endoscopically active was defined as SES-CD > 3 for CD and UCEIS >1 for UC. Previous surgery included any resection of a part of the gut, stricturoplasty, and fistulectomy or fistulotomy. Patients’ treatment regimens were recorded after admission, including 5-aminosalicylate acid (5-ASA), corticosteroids, azathioprine/6-mercaptopurine (AZA/6-MP), methotrexate, biological agents (infliximab, adalimumab, ustekinumab, and vedolizumab) and enteral nutrition. Subsequent clinical and treatment data were collected during the follow-up medical visits. Clinicians who followed patients were blinded to the results of the patients’ HADS scores at the time of recruitment. The study endpoints included the occurrence of treatment non-response and IBD-related poor outcomes. A flowchart of the study design was shown in Figure 1.

### 2.2. Treatment efficacy and IBD-related poor outcomes measurement

Steroid resistance was identified in patients who had no disease remission within the 4 weeks of treatment with the equivalent of 40–60 mg (0.75–1 mg/kg) prednisone daily, or as a result of a transition to alternative medical therapy (i.e., biologicals or cyclosporine) or required surgery during this period (23, 27). Resistance to AZA/6-MP was identified as a lack of clinical improvement or relapse despite thiopurines at an appropriate dose for at least 3 months (i.e., azathioprine 2–2.5 mg/kg/day or mercaptopurine 1–1.5 mg/kg/day in the absence of leukopenia) (27). Primary nonresponse to anti-TNF agents was defined as a lack of clinical improvement by 12 weeks after starting therapy accompanied by an alteration of therapeutic approach (addition or escalation of corticosteroids, switch to a different agent, or surgery) (28). IBD-related poor outcomes, including IBD-related hospitalization, IBD-related resective surgery, or death. IBD-related hospitalization was an unscheduled admission including an overnight stay with a diagnosis of CD or UC as the most responsible or primary diagnosis.

### 2.3. Outcomes

The study outcomes were to investigate the association between anxiety/depression symptoms and (1) the nonresponse to medications, including corticosteroids, AZA/6-MP, and anti-TNF agents, as well as (2) the occurrence of IBD-related poor outcomes defined above. Patients who were selected as a subgroup to analyze the efficacy of a medications have not used it in the latest 3 months before enrollment. Corticosteroids, AZA/6-MP, and anti-TNF agents were chosen because they are the most commonly used drugs in our hospital for the treatment of IBD patients.

### 2.4. Sample size

The sample size was calculated using the PASS15 software package. A power analysis was performed using *α* = 0.05 and *β* = 0.80. Based on the results of our pre-test, the rates of steroid resistance in patients with and without anxiety and/or depression symptoms were 0.45 and 0.2, respectively. The required sample size in the corticosteroids subgroup was determined to be 108. According to two large observational studies (13, 15), we assumed the frequencies of disease-related poor outcomes of the two groups are 29 and 16%. Overall, 317 patients were required. A dropout rate of approximately 10% was predicted. Therefore, we planned to include more than 349 patients.

### 2.5. Statistical analysis

Continuous variables are reported as medians with interquartile ranges (IQRs) and categorical variables are reported as numbers with percentages. Mann–Whitney U test and chi-square tests/Fisher’s exact test were used for the comparison of continuous and categorical variables, respectively. The correlation of HADS anxiety and depression scores was calculated by Pearson correlation analysis. Variables that were considered clinically relevant or that had a *p* value less than 0.1 in the univariate analysis were selected for the subsequent binary logistic regression and a stepwise procedure (forward: LR) was used to determine independent influencing factors of therapeutic efficacy. Kaplan–Meier curves showing the cumulative incidence of poor outcomes in patients with and without anxiety/depression symptoms were compared by log-rank test. The Cox regression model was applied to investigate the association between anxiety/depression symptoms and poor outcomes by controlling for baseline demographic characteristics, type of IBD, disease duration, disease activity, and previous surgery. Besides, we performed Mantel–Haenszel *χ*^2^ test to explore whether there was a linear relationship between the HADS score and the occurrence of poor outcomes. A two-tailed *p* value less than.05 was considered significant. All statistical analyses were performed using IBM SPSS 22.0 (SPSS, Inc., Chicago, IL) and GraphPad Prism 8.0 software packages.

## 3. Results

### 3.1. Baseline characteristics

In this study, a total of 325 IBD patients, including 89 UC and 236 CD patients were finally enrolled. The median follow-up time was 18.63 months (IQR: 10.05–25.93) since HADS was completed. Baseline characteristics are shown in Table 1. The mean age of the patients was 33 ± 13 years, and 212 (65.2%) were male. Nearly 30.8% had anxiety symptoms, and 31.4% had depression symptoms.

**Table 1:** Demographic characteristics.

	Without symptoms of anxiety/depression (N = 201)	With symptoms of anxiety/depression (N = 124)	*p* value
Age (years)	28 (22–39)	30 (24–45)	**0.030**
Gender (male)	134 (66.7%)	78 (62.9%)	0.489
BMI	18.6 (17.1–20.7)	18.3 (16.7–21.1)	0.748
Tobacco use	35 (17.4%)	23 (18.5%)	0.795
Disease type (UC)	54 (26.9%)	35 (28.2%)	0.789
Disease duration (months)	26.5 (8–72)	29 (12–77)	0.365
MAYO	10 (8–11)	11 (10–11)	0.116
CDAI	217 (141–268)	268 (191–341)	**<0.001**
Clinical active	160 (79.6%)	113 (91.1%)	**0.006**
Previous surgery	47 (23.4%)	33 (26.6%)	0.511
Initial CD location			
L1 (ileum)	16 (11.0%)	7 (8.0%)	0.571
L2 (colon)	22 (15.2%)	17 (19.5%)	
L3 (ileum and colon)	107 (73.8%)	63 (72.4%)	
Missing or unknown	2	2	
Initial UC location			
E1 (Proctitis)	6 (12.0%)	3 (8.6%)	0.541
E2 (Left-sided colitis)	7 (14.0%)	8 (22.8%)	
E3 (Pancolitis)	37 (74.0%)	24 (68.6%)	
Missing or unknown	4	0	
SES-CD	11 (7–15)	13 (9–18)	**0.016**
UCEIS	4 (3–5)	5 (4–6)	**0.028**
Laboratory studies			
Hemoglobin (g/L)	115 (96–132)	109 (88–129)	0.076
WBC (10^9^/L)	6.40 (4.94–8.30)	6.35 (4.94–9.01)	0.754
Platelets (10^9^/L)	285 (220–359)	303 (233–431)	0.084
CRP (mg/L)	13.4 (3.5–36.4)	19.2 (5.27–51.1)	0.110
ESR (mm/h)	37 (18–66)	49 (27–72)	**0.009**
Albumin (g/L)	37.9 (33.7–43.1)	36.1 (31.8–43.2)	0.078
EBV-DNA-positive	17 (9.6%)	18 (15.7%)	0.120
CMV-DNA-positive	23 (13.0%)	18 (15.7%)	0.523
Treatment within 3 months before enrollment			
5-aminosalicylate acid	71 (35.3%)	40 (32.3%)	
Immunosuppressors	25 (12.4%)	20 (16.1%)	
Antibiotics	12 (0.06%)	9 (0.07%)	
Enteral nutrition	3 (0.01%)	0 (0%)	

BMI, body mass index; UC, ulcerative colitis; CD, Crohn’s disease; SES-CD, Simple Endoscopic Score for Crohn’s disease; UCEIS ulcerative colitis endoscopic index of severity; WBC, white blood cells; CRP, C-reactive protein; ESR, erythrocyte sedimentation rate; EBV, Epstein–Barr virus; CMV, cytomegalovirus. The bold values indicate *p*-values less than 0.05.

The median age of IBD patients with anxiety/depressive symptoms was 30 (IQR: 24–45), which was older than the median age of 28 (IQR: 22–39) of those without (*p* = 0.030). In contrast to patients without anxiety/depression symptoms, those with anxiety/depression symptoms were more likely to be in the disease active stage (*p* = 0.006) and had higher SES-CD/UCEIS (*p* = 0.016, *p* = 0.028, respectively), and higher ESR levels (*p* = 0.009). However, there were no statistically significant differences in gender, BMI, tobacco use, or other clinical features between the groups of IBD patients with and without anxiety/depression symptoms.

We also assessed the correlation of the HADS-A and HADS-D score, and the results showed that the two variables had a strong correlation (*r* = 0.767, 95% CI: 0.718–0.808, *p* < 0.001, Supplementary Figure S1). Seventy-eight percent of IBD patients who reported having anxiety symptoms (HADS-A ≥ 8) also reported having depressive symptoms (HADS-D ≥ 8). Similarly, 76.5% of those with symptoms of depression (HADS-D ≥ 8) were accompanied by symptoms of anxiety (HADS-A ≥ 8). As a result, IBD patients with anxiety and/or depressive symptoms were placed in the same group.

### 3.2. Symptoms of anxiety/depression related to glucocorticoid resistance

**Figure 2:**
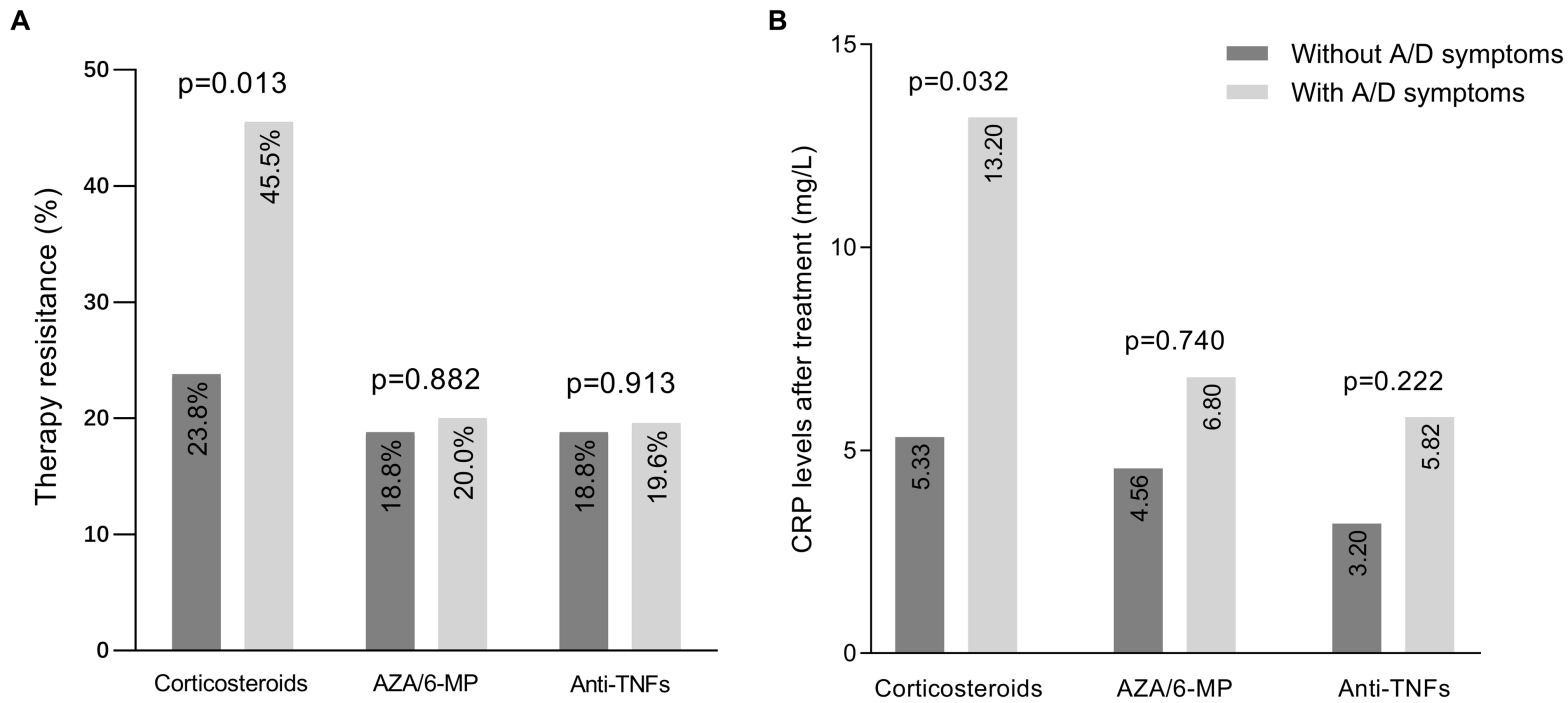
Efficacy of medications in IBD patients with and without anxiety/depression symptoms. **(A)** Prevalence of resistance to three medications in patients with and without symptoms of anxiety/depression. **(B)** CRP levels after one month of corticosteroids therapy, three months of AZA/6-MP therapy, and three months of anti-TNF agents therapy. IBD, inflammatory bowel disease; A/D, anxiety/depression; AZA/6-MP, azathioprine/6-mercaptopurine.

Of the 325 IBD patients enrolled, 118 were treated with corticosteroids, 88 with AZA/6-MP, and 147 with anti-TNF agents, after completing HADS. Firstly, we assessed the association between anxiety/depression symptoms and the therapeutic efficacy of these three medications (Figure 2A). In patients with anxiety/depression symptoms, there were 25 (45.5%) cases of steroid resistance, 8 (20%) cases of AZA/6-MP resistance, and 9 (19.6%) cases of primary nonresponse to anti-TNF drugs, all of which were higher than those without anxiety/depression symptoms. However, only steroid resistance was shown to be statistically different between the two groups (*p* = 0.013), while resistance to AZA/6-MP (*p* = 0.882) and anti-TNF agents (*p* = 0.913) were not. We also compared the post-treatment CRP levels of patients in each group (Figure 2B). After receiving corticosteroids treatment for one month, CRP levels in IBD patients with anxiety/depression symptoms were considerably higher than those without (*p* = 0.032). After 3 months of AZA/6-MP and anti-TNF agents treatment, the differences in CRP levels between the two groups were not statistically significant (*p* = 0.740 and *p* = 0.222, respectively).

Secondly, we applied logistic regression analysis to determine the relationship between anxiety/depression symptoms and steroid resistance. In the univariate logistic regression analysis, anxiety/depression symptoms, BMI, concomitant use of immunosuppressors, and CMV-DNA positive were associated with steroid resistance (*p* = 0.014, *p* = 0.014, *p* = 0.029, *p* = 0.036, respectively, Table 2). In multivariate analysis, coexisting with anxiety/depression symptoms was independently associated with steroid resistance (OR: 3.047, 95% CI: 1.186–7.824, *p* = 0.021, Table 2) when adjusting for covariates. We also used multivariate-adjusted models to investigate whether anxiety/depression symptoms were associated with the efficacy of AZA/6-MP and anti-TNF agents. The data revealed no significant correlation between anxiety/depression symptoms and AZA/6-MP resistance, or anti-TNF agents nonresponse, even after adjusting for age, sex, disease severity, and other covariates (Supplementary Table S1).

**Table 2:** Univariate and multivariate analysis of influencing factors for steroid resistance.

Variables	Univariate analysis		Multivariate analysis	
	OR (95% CI)	*p* value	OR (95% CI)	*p* value
With anxiety/depression symptoms	2.667 (1.215–5.852)	**0.014**	3.047 (1.186–7.824)	**0.021**
Age (year)	1.020 (0.995–1.046)	0.122		
Gender (male)	0.730 (0.327–1.628)	0.442		
BMI	1.184 (1.035–1.355)	**0.014**	1.128 (0.973–1.307)	0.110
Tobacco use	1.143 (0.435–3.005)	0.787		
Disease type (UC)	0.702 (0.326–1.514)	0.367		
Disease Duration (month)	0.998 (0.990–1.007)	0.998		
Previous surgery	0.321 (0.067–1.523)	0.152		
Clinical active	1.750 (0.201–15.262)	0.613		
Endoscopic active	-	0.999		
Concomitant use of 5-ASA	1.200 (0.402–3.579)	0.744		
Concomitant use of IMM	0.417 (0.191–0.913)	**0.029**	0.663 (0.238–1.846)	0.432
CRP levels	1.001 (0.993–1.008)	0.849		
ESR levels	1.002 (0.990–1.015)	0.700		
Albumin levels	0.969 (0.907–1.035)	0.346		
EBV-DNA positive	2.231 (0.885–5.621)	0.089	1.227 (0.399–3.767)	0.721
CMV-DNA positive	2.634 (1.104–6.282)	**0.036**	2.644 (0.893–7.825)	0.079

BMI, body mass index; UC, ulcerative colitis; 5-ASA, 5-aminosalicylic acid; IMM, immunosuppressor; CRP, C-reactive protein; ESR, erythrocyte sedimentation rate; EBV, Epstein–Barr virus; CMV, cytomegalovirus; OR, odds ratio; CI, confidence interval. The bold values indicate *p*-values less than 0.05.

### 3.3. Symptoms of anxiety/depression related to poor outcomes

**Figure 3:**
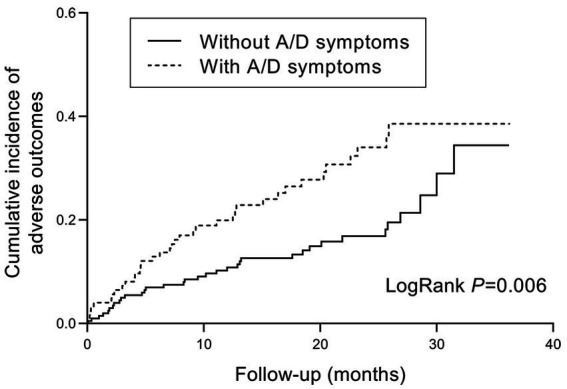
Kaplan–Meier hazard curve for IBD-related poor outcomes in patients with and without anxiety/depression symptoms. IBD, inflammatory bowel disease; A/D, anxiety/depression.

**Figure 4:**
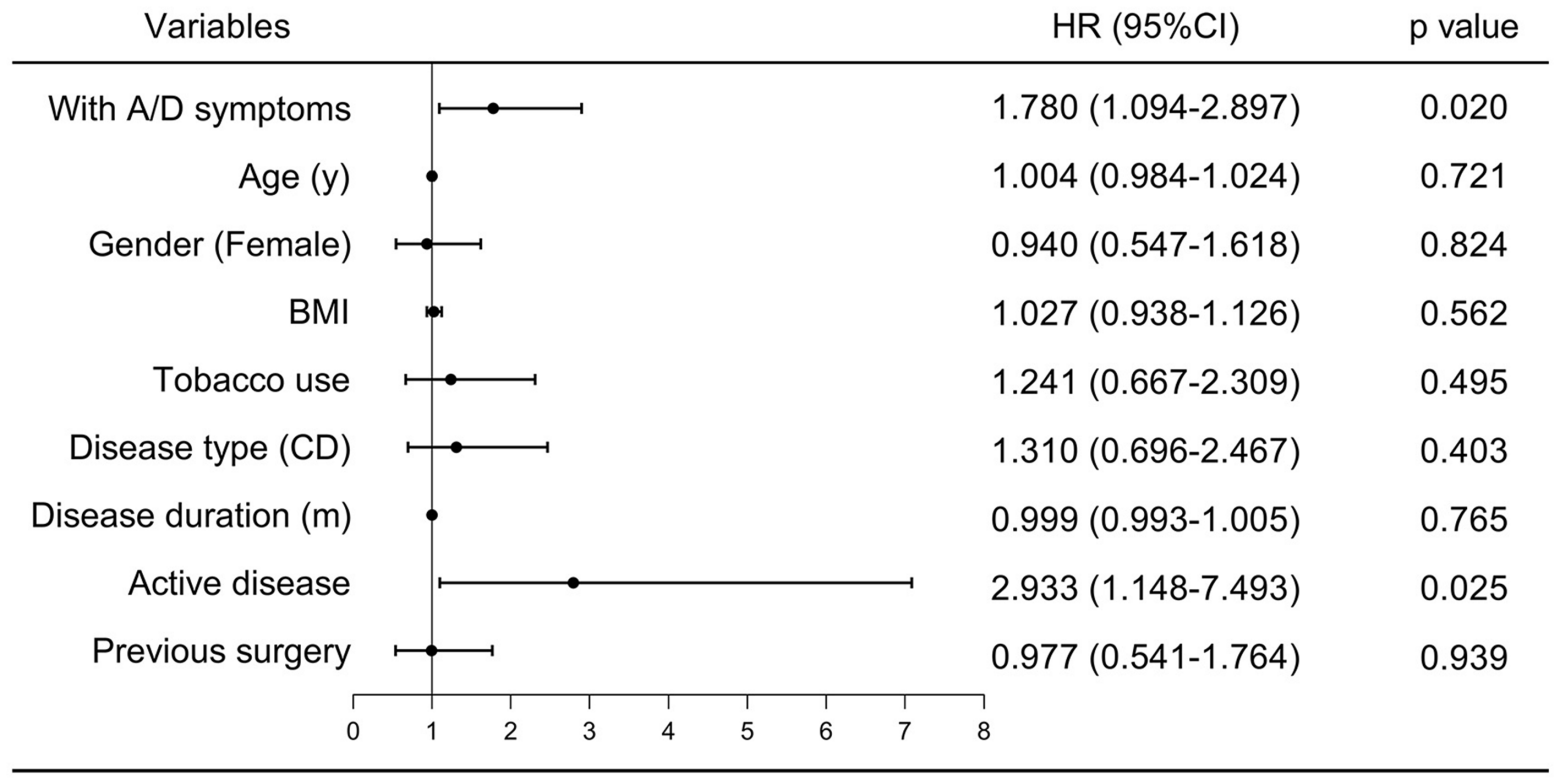
Multivariate Cox Regression Analysis of influencing factors for IBD-related poor outcomes. A/D, anxiety/depression; BMI, body mass index; UC, ulcerative colitis; HR, hazard ratio; CI, confidence interval.

During the follow-up period, poor outcomes occurred in 37/124 (29.8%) of patients with anxiety/depression symptoms, and 35/201 (17.4%) of patients without. The cumulative incidence of poor outcomes was significantly higher in patients with anxiety/depression symptoms (log-rank test, *p* = 0.006, Figure 3). In the Cox regression model, the crude hazard ratio (HR) of anxiety/depression symptoms for poor outcomes was 1.898 (95% CI: 1.195–3.014, *p* = 0.007). After adjustment for demographic and clinical covariates, including age, gender, BMI, tobacco use, disease type, disease duration, and previous surgery, the HR of anxiety/depression symptoms for poor outcomes remained significant (HR: 1.780, 95% CI: 1.094–2.897, *p* = 0.020, Figure 4). Except for anxiety/depression symptoms, active disease was also an independent predictive factor for the incidence of poor outcomes.

**Figure 5:**
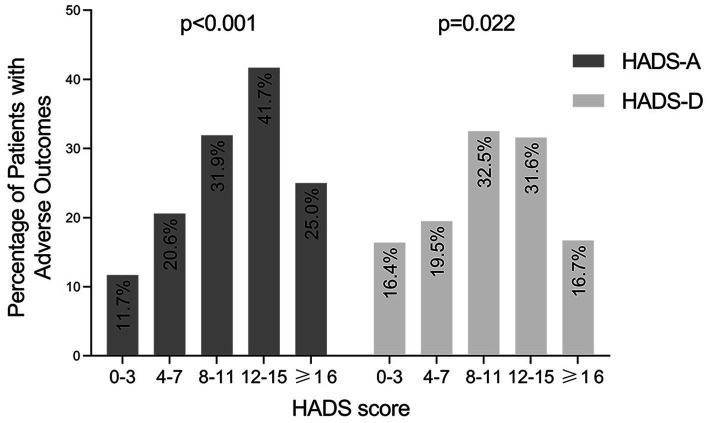
Occurrence of IBD-related adverse outcomes in patients grouped by HADS-A and HADS-D score. IBD, inflammatory bowel disease; HADS-A, the anxiety score of the Hospital Anxiety and Depression Scale; HADS-D, the depression score of the Hospital Anxiety and Depression Scale.

In addition, we also used Mantel–Haenszel χ2 test to evaluate whether IBD-related poor outcomes were more likely to be seen in patients with increased anxiety or depression scores. As shown in Figure 5, with HADS-A and HADS-D score increased, more patients experienced IBD-related poor outcomes. The Mantel–Haenszel *χ*^2^ test revealed that there was a linear relationship between HADS-A and HADS-D scores and IBD-related poor outcomes (*p* < 0.001 and *p* = 0.022, respectively).

## 4. Discussion

This study is the first longitudinal observational study to assess the influence of anxiety/depression symptoms on drug efficacy in IBD. The results showed that concomitant anxiety/depression symptoms was an independent influencing factor for the development of steroid resistance and disease-related poor outcomes in IBD patients, including hospitalization, resective surgery, and death. A recent meta-analysis showed that the prevalence of anxiety and depression symptoms in IBD patients was 33.2 and 21.6%, respectively, based on the HADS scale (3). According to our findings, 30.8% of IBD patients exhibited anxiety symptoms, and 31.4% had depression symptoms. Such differences could be attributable to the inconsistent baseline characteristics of patients between the two studies. Our results further demonstrated that anxiety and depression symptoms are more common in IBD patients with active disease, which was consistent with previous findings (29).

The reason why patients with anxiety/depression symptoms are more likely to develop steroid resistance, as shown in our study, is not yet clear. Corticosteroids mediate their anti-inflammatory responses by binding to the intracellular GR (30, 31). A condition of glucocorticoid resistance could result from a damaged GR, whether as a result of decreased expression, decreased binding affinity to its ligand, nuclear translocation, DNA binding, or interaction with other transcription factors (such as NF-B, AP-1) (19). Decreased GR mRNA expression was reported in patients with depression and post-traumatic stress disorder (32, 33). Coincidentally, GR mRNA expression was also decreased in patients with steroid resistance in IBD and other diseases (34–36). Moreover, depressed patients were reported to have higher FK506 binding protein 5 (FKBP5) mRNA expression (32), which is considered to be a negative regulator of glucocorticoid action and reduces GR binding affinity (37, 38). These may be the crosstalk between depression/anxiety and steroid resistance. UCEIS and fecal calprotectin have been reported to be associated with steroid nonresponse in patients with UC (39). But in our study, the endoscopically active was not associated with steroid resistance. Additionally, it has been reported that weight loss in UC patients is related to steroid refractoriness (40). Similarly, in the univariate analysis of our study, lower BMI was linked to steroid resistance. However, the relationship was no longer significant in the multivariate analysis.

Besides, we did not find a correlation between anxiety/depression symptoms and the efficacy of AZA/6-MP and anti-TNF agents. A previous study also found that HADS-A ≥ 8 did not affect the response to infliximab (41), which is consistent with our findings. For AZA or 6-MP, previous studies have reported several genes such as Glutathione-S-Transferase M1(GSTM1) (42, 43), and Inosine triphosphate pyrophosphatase (ITPA) (44), that are related to thiopurines nonresponse. However, the accurate predictors of thiopurine nonresponse have not been confirmed. Additionally, no previous research has explored the relationship between psychiatric disorders and their efficacy. Therefore, more research is needed to confirm these findings.

The relationship between mood disorders and disease outcomes in IBD is controversial. A large cohort study of 2,289 IBD patients suggested a significant association between anxiety/depression symptoms and clinical recurrence (13). Also, another smaller study showed that anxiety symptom, instead of depression symptom, was the independent predictor of IBD-related poor outcomes, such as emergency room visits, hospitalization, and requiring systemic steroids (45). On the other hand, another study reported contrary results that the risk of developing poor outcomes was not significantly different between patients with and without anxiety and mood disorders (17). According to our findings, the presence of anxiety/depression symptoms and active disease were independent influencing factors of IBD-related poor outcomes, including urgent IBD-related hospitalization, IBD-related intestinal resection, and death. It is possible that the different criteria and timing for assessing anxiety/depression symptoms have led to different results. In our study, patients with anxiety/depression symptoms were more likely to be in the active stage of IBD. However, the association between anxiety/depression symptoms and disease-related poor outcomes existed even adjusted for disease activity. As previously reported, in patients with quiescent IBD at baseline, abnormal anxiety scores were related to later relapse (46).

Furthermore, we also found a linear relationship between HADS-A and HADS-D scores and poor outcomes. It was similar to the report by Narula (45), but they only assessed the linear relationship between HADS-A and poor outcomes. We used HADS as a screening instrument to measure anxiety and depression symptoms in IBD patients. Although it was not designed to provide an actual diagnosis of these disorders, it has been used as a screening tool for anxiety and depression symptoms in some diseases and has been frequently used in IBD studies (7, 13, 45–47). We grouped IBD patients with anxiety and/or depression symptoms together, due to the strong correlations between HADS-A and HADS-D scores (48). Additionally, the comorbidity between anxiety and depression was common. Approximately 85% of patients with depression have significant anxiety symptoms, and 90% of patients with anxiety disorder have comorbid depression (49).

There are several strengths of this study. We prospectively collected patients’ data from enrollment and follow-up, including the use of the HADS questionnaire to identify their anxiety/depression symptoms at the time of enrollment. Previous studies have rarely explored the association between psychiatric comorbidity and treatment efficacy in IBD patients. As far as we know, this is the first study to demonstrate a strong association between anxiety/depression symptoms and steroid resistance in IBD patients. Limitations of this study include that this was a single-center study, and due to the limited sample size, CD and UC were analyzed together as IBD to have sufficient power to determine predictors.

## 5. Conclusion

These findings highlight the importance of recognizing the impact of psychiatric disorders on IBD patients, because these patients may be at a higher risk of developing steroid resistance and IBD-related poor outcomes. Further studies are needed to explore why patients with anxiety/depression symptoms have higher rates of steroid resistance, and whether interventions for anxiety and depression alter their response to medications and improve their prognosis.

## Data availability statement

The original contributions presented in the study are included in the article/Supplementary material, further inquiries can be directed to the corresponding author.

## Ethics statement

The studies involving human participants were reviewed and approved by Ethics Committee of West China Hospital of Sichuan University. The patients/participants provided their written informed consent to participate in this study.

## Author contributions

SD, YY, and YC: study concept and design, acquisition of data, analysis and interpretation of data, drafting of the manuscript, critical revision of the manuscript for important intellectual content, and statistical analysis. PC: acquisition of data, analysis, and interpretation of data. CL: acquisition of data. YZ: securing funding, technical or material support, study supervision, and critical revision of the manuscript for important intellectual content. All authors have approved the final draft submitted.

## Funding

This work was supported by National Natural Science Foundation of China (No: 81770550), the Major Science and Technology Projects in Sichuan Province (No: 2021YFS0149), 1·3·5 Project for Disciplines of Excellence, West China Hospital, Sichuan University (No: ZYJC21044).

## Conflict of interest

The authors declare that the research was conducted in the absence of any commercial or financial relationships that could be construed as a potential conflict of interest.

## Publisher’s note

All claims expressed in this article are solely those of the authors and do not necessarily represent those of their affiliated organizations, or those of the publisher, the editors and the reviewers. Any product that may be evaluated in this article, or claim that may be made by its manufacturer, is not guaranteed or endorsed by the publisher.

## References

[1] Frolkis AD Vallerand IA Shaheen AA Lowerison MW Swain MG Barnabe C. Depression increases the risk of inflammatory bowel disease, which may be mitigated by the use of antidepressants in the treatment of depression. Gut. (2019) 68:1606–12. doi: 10.1136/gutjnl-2018-317182 30337374

[2] Blackwell J Saxena S Petersen I Hotopf M Creese H Bottle A. Depression in individuals who subsequently develop inflammatory bowel disease: a population-based nested case-control study. Gut. (2021) 70:1642–8. doi: 10.1136/gutjnl-2020-322308, PMID: 33109601

[3] Barberio B Zamani M Black CJ Savarino EV Ford AC. Prevalence of symptoms of anxiety and depression in patients with inflammatory bowel disease: a systematic review and Meta-analysis. Lancet Gastroenterol Hepatol. (2021) 6:359–70. doi: 10.1016/s2468-1253(21)00014-5 33721557

[4] Neuendorf R Harding A Stello N Hanes D Wahbeh H. Depression and anxiety in patients with inflammatory bowel disease: a systematic review. J Psychosom Res. (2016) 87:70–80. doi: 10.1016/j.jpsychores.2016.06.001, PMID: 27411754

[5] Choi K Chun J Han K Park S Soh H Kim J. Risk of anxiety and depression in patients with inflammatory bowel disease: a Nationwide, population-based study. J Clin Med. (2019) 8:654. doi: 10.3390/jcm8050654, PMID: 31083476 PMC6572298

[6] Porcelli P Leoci C Guerra V. A prospective study of the relationship between disease activity and psychologic distress in patients with inflammatory bowel disease. Scand J Gastroenterol. (1996) 31:792–6. doi: 10.3109/00365529609010354, PMID: 8858749

[7] Navabi S Gorrepati VS Yadav S Chintanaboina J Maher S Demuth P. Influences and impact of anxiety and depression in the setting of inflammatory bowel disease. Inflamm Bowel Dis. (2018) 24:2303–8. doi: 10.1093/ibd/izy143, PMID: 29788469

[8] Romberg-Camps MJ Dagnelie PC Kester AD Hesselink-van de Kruijs MA Cilissen M Engels LG. Influence of phenotype at diagnosis and of other potential prognostic factors on the course of inflammatory bowel disease. Am J Gastroenterol. (2009) 104:371–83. doi: 10.1038/ajg.2008.38 19174787

[9] Beaugerie L Seksik P Nion-Larmurier I Gendre JP Cosnes J. Predictors of Crohn's disease. Gastroenterology. (2006) 130:650–6. doi: 10.1053/j.gastro.2005.12.019 16530505

[10] Bessissow T Lemmens B Ferrante M Bisschops R Van Steen K Geboes K. Prognostic value of serologic and histologic markers on clinical relapse in ulcerative colitis patients with mucosal healing. Am J Gastroenterol. (2012) 107:1684–92. doi: 10.1038/ajg.2012.301, PMID: 23147523

[11] Bitton A Peppercorn MA Antonioli DA Niles JL Shah S Bousvaros A. Clinical, biological, and histologic parameters as predictors of relapse in ulcerative colitis. Gastroenterology. (2001) 120:13–20. doi: 10.1053/gast.2001.20912, PMID: 11208709

[12] Kochar B Barnes EL Long MD Cushing KC Galanko J Martin CF. Depression is associated with more aggressive inflammatory bowel disease. Am J Gastroenterol. (2018) 113:80–5. doi: 10.1038/ajg.2017.423, PMID: 29134965 PMC5962285

[13] Mikocka-Walus A Pittet V Rossel JB von Kanel R Swiss IBDCSG. Symptoms of depression and anxiety are independently associated with clinical recurrence of inflammatory bowel disease. Clin Gastroenterol Hepatol. (2016) 14:829–835.e1. doi: 10.1016/j.cgh.2015.12.045, PMID: 26820402

[14] Fairbrass KM Gracie DJ Ford AC. Longitudinal follow-up study: effect of psychological co-morbidity on the prognosis of inflammatory bowel disease. Aliment Pharmacol Ther. (2021) 54:441–50. doi: 10.1111/apt.16454 34114664

[15] Ananthakrishnan AN Gainer VS Perez RG Cai T Cheng SC Savova G. Psychiatric co-morbidity is associated with increased risk of surgery in Crohn's disease. Aliment Pharmacol Ther. (2013) 37:445–54. doi: 10.1111/apt.12195 23289600 PMC3552092

[16] Fairbrass KM Lovatt J Barberio B Yuan Y Gracie DJ Ford AC. Bidirectional brain-gut Axis effects influence mood and prognosis in Ibd: a systematic review and Meta-analysis. Gut. (2022) 71:1773–80. Epub 20211101. doi: 10.1136/gutjnl-2021-325985, PMID: 34725197

[17] Dolovich C Bernstein CN Singh H Nugent Z Tennakoon A Shafer LA. Anxiety and depression leads to anti-tumor necrosis factor discontinuation in inflammatory bowel disease. Clin Gastroenterol Hepatol. (2021) 19:1200–1208.e1. doi: 10.1016/j.cgh.2020.07.013 32668341

[18] Gracie DJ Hamlin PJ Ford AC. The influence of the brain–gut Axis in inflammatory bowel disease and possible implications for treatment. Lancet Gastroenterol Hepatol. (2019) 4:632–42. doi: 10.1016/s2468-1253(19)30089-5 31122802

[19] Silverman MN Sternberg EM. Glucocorticoid regulation of inflammation and its functional correlates: from Hpa Axis to glucocorticoid receptor dysfunction. Ann N Y Acad Sci. (2012) 1261:55–63. doi: 10.1111/j.1749-6632.2012.06633.x, PMID: 22823394 PMC3572859

[20] Fiksdal A Hanlin L Kuras Y Gianferante D Chen X Thoma MV. Associations between symptoms of depression and anxiety and cortisol responses to and recovery from acute stress. Psychoneuroendocrinology. (2019) 102:44–52. doi: 10.1016/j.psyneuen.2018.11.035, PMID: 30513499 PMC6420396

[21] Sidoroff M Kolho KL. Glucocorticoid sensitivity in inflammatory bowel disease. Ann Med. (2012) 44:578–87. doi: 10.3109/07853890.2011.590521 21992564

[22] Derijks LJJ Wong DR Hommes DW van Bodegraven AA. Clinical pharmacokinetic and Pharmacodynamic considerations in the treatment of inflammatory bowel disease. Clin Pharmacokinet. (2018) 57:1075–106. doi: 10.1007/s40262-018-0639-4 29512050

[23] Gomollon F Dignass A Annese V Tilg H Van Assche G Lindsay JO. 3rd European evidence-based consensus on the diagnosis and Management of Crohn's disease 2016: part 1: diagnosis and medical management. J Crohns Colitis. (2017) 11:3–25. doi: 10.1093/ecco-jcc/jjw168, PMID: 27660341

[24] Magro F Gionchetti P Eliakim R Ardizzone S Armuzzi A Barreiro-de Acosta M. Third European evidence-based consensus on diagnosis and Management of Ulcerative Colitis. Part 1: definitions, diagnosis, extra-intestinal manifestations, pregnancy, Cancer surveillance, surgery, and Ileo-anal pouch disorders. J Crohns Colitis. (2017) 11:649–70. doi: 10.1093/ecco-jcc/jjx008, PMID: 28158501

[25] D'Haens G Sandborn WJ Feagan BG Geboes K Hanauer SB Irvine EJ. A review of activity indices and efficacy end points for clinical trials of medical therapy in adults with ulcerative colitis. Gastroenterology. (2007) 132:763–86. doi: 10.1053/j.gastro.2006.12.038, PMID: 17258735

[26] Nagy Z Nagy A Karadi O Figler M Rumi G Suto G. Prevalence of the factor V Leiden mutation in human inflammatory bowel disease with different activity. J Physiol Paris. (2001) 95:483–7. doi: 10.1016/s0928-4257(01)00067-5, PMID: 11595479

[27] Dignass A Eliakim R Magro F Maaser C Chowers Y Geboes K. Second European evidence-based consensus on the diagnosis and Management of Ulcerative Colitis Part 1: definitions and diagnosis. J Crohns Colitis. (2012) 6:965–90. doi: 10.1016/j.crohns.2012.09.003, PMID: 23040452

[28] Barber GE Yajnik V Khalili H Giallourakis C Garber J Xavier R. Genetic markers predict primary non-response and durable response to anti-Tnf biologic therapies in Crohn's disease. Am J Gastroenterol. (2016) 111:1816–22. doi: 10.1038/ajg.2016.408, PMID: 27596696 PMC5143156

[29] Tribbick D Salzberg M Ftanou M Connell WR Macrae F Kamm MA. Prevalence of mental health disorders in inflammatory bowel disease: an Australian outpatient cohort. Clin Exp Gastroenterol. (2015) 8:197–204. doi: 10.2147/ceg.S77567 26213474 PMC4512611

[30] Barnes PJ Adcock IM. Glucocorticoid resistance in inflammatory diseases. Lancet. (2009) 373:1905–17. doi: 10.1016/s0140-6736(09)60326-3 19482216

[31] Farrell RJ Kelleher D. Glucocorticoid resistance in inflammatory bowel disease. J Endocrinol. (2003) 178:339–46. doi: 10.1677/joe.0.1780339 12967327

[32] Cattaneo A Ferrari C Turner L Mariani N Enache D Hastings C. Whole-blood expression of Inflammasome-and glucocorticoid-related Mrnas correctly separates treatment-resistant depressed patients from drug-free and responsive patients in the biodep study. Transl Psychiatry. (2020) 10:232. doi: 10.1038/s41398-020-00874-7, PMID: 32699209 PMC7376244

[33] Gola H Engler A Morath J Adenauer H Elbert T Kolassa IT. Reduced peripheral expression of the glucocorticoid receptor alpha isoform in individuals with posttraumatic stress disorder: a cumulative effect of trauma burden. PLoS One. (2014) 9:e86333. doi: 10.1371/journal.pone.0086333, PMID: 24466032 PMC3897679

[34] Raddatz D Middel P Bockemuhl M Benohr P Wissmann C Schworer H. Glucocorticoid receptor expression in inflammatory bowel disease: evidence for a mucosal Down-regulation in steroid-unresponsive ulcerative colitis. Aliment Pharmacol Ther. (2004) 19:47–61. doi: 10.1046/j.1365-2036.2003.01802.x, PMID: 14687166

[35] Ma LL Fang MY Liang Y Xiang Y Jia ZL Sun XL. Low expression of glucocorticoid receptor alpha isoform in adult immune thrombocytopenia correlates with glucocorticoid resistance. Ann Hematol. (2013) 92:953–60. doi: 10.1007/s00277-013-1705-5 23435844

[36] Sun XL Fang MY Guan YC Si Y Ma LL Wang Y. Changes of glucocorticoid receptor isoforms expression in acute lymphoblastic leukemia correlate with glucocorticoid resistance. Pharmazie. (2015) 70:316–21. doi: 10.1691/ph.2015.4813 26062300

[37] De Iudicibus S Franca R Martelossi S Ventura A Decorti G. Molecular mechanism of glucocorticoid resistance in inflammatory bowel disease. World J Gastroenterol. (2011) 17:1095–108. doi: 10.3748/wjg.v17.i9.1095 21448414 PMC3063901

[38] Wochnik GM Ruegg J Abel GA Schmidt U Holsboer F Rein T. Fk506-binding proteins 51 and 52 differentially regulate dynein interaction and nuclear translocation of the glucocorticoid receptor in mammalian cells. J Biol Chem. (2005) 280:4609–16. doi: 10.1074/jbc.M407498200, PMID: 15591061

[39] Xie T Zhao C Ding C Zhang T Dai X Lv T. Fecal calprotectin as an alternative to ulcerative colitis endoscopic index of severity to predict the response to corticosteroids of acute severe ulcerative colitis: a prospective observational study. Dig Liver Dis. (2017) 49:984–90. doi: 10.1016/j.dld.2017.04.021 28539226

[40] Li J Wang F Zhang HJ Sheng JQ Yan WF Ma MX. Corticosteroid therapy in ulcerative colitis: clinical response and predictors. World J Gastroenterol. (2015) 21:3005–15. doi: 10.3748/wjg.v21.i10.3005, PMID: 25780299 PMC4356921

[41] Persoons P Vermeire S Demyttenaere K Fischler B Vandenberghe J Van Oudenhove L. The impact of major depressive disorder on the short-and Long-term outcome of Crohn's disease treatment with infliximab. Aliment Pharmacol Ther. (2005) 22:101–10. doi: 10.1111/j.1365-2036.2005.02535.x 16011668

[42] Stocco G Cuzzoni E De Iudicibus S Franca R Favretto D Malusa N. Deletion of glutathione-S-transferase M1 reduces azathioprine metabolite concentrations in young patients with inflammatory bowel disease. J Clin Gastroenterol. (2014) 48:43–51. doi: 10.1097/MCG.0b013e31828b2866, PMID: 23787247

[43] Lucafo M Stocco G Martelossi S Favretto D Franca R Malusa N. Azathioprine biotransformation in young patients with inflammatory bowel disease: contribution of glutathione-S transferase M1 and A1 variants. Genes. (2019) 10:277. doi: 10.3390/genes10040277, PMID: 30987408 PMC6523194

[44] Zabala-Fernandez W Barreiro-de Acosta M Echarri A Carpio D Lorenzo A Castro J. A pharmacogenetics study of Tpmt and Itpa genes detects a relationship with side effects and clinical response in patients with inflammatory bowel disease receiving azathioprine. J Gastrointestin Liver Dis. (2011) 20:247–53. PMID: 21961091

[45] Narula N Pinto-Sanchez MI Calo NC Ford AC Bercik P Reinisch W. Anxiety but not depression predicts poor outcomes in inflammatory bowel disease. Inflamm Bowel Dis. (2019) 25:1255–61. doi: 10.1093/ibd/izy385, PMID: 30615113

[46] Gracie DJ Guthrie EA Hamlin PJ Ford AC. Bi-directionality of brain-gut interactions in patients with inflammatory bowel disease. Gastroenterology. (2018) 154:1635–1646.e3. doi: 10.1053/j.gastro.2018.01.027 29366841

[47] Marrie RA Graff LA Fisk JD Patten SB Bernstein CN. The relationship between symptoms of depression and anxiety and disease activity in Ibd over time. Inflamm Bowel Dis. (2021) 27:1285–93. doi: 10.1093/ibd/izaa349, PMID: 33393632 PMC8314114

[48] Bjelland I Dahl AA Haug TT Neckelmann D. The validity of the hospital anxiety and depression scale – an updated literature review. J Psychosom Res. (2002) 52:69–77. doi: 10.1016/s0022-3999(01)00296-3 11832252

[49] Tiller JWG. Depression and anxiety. Med J Aust. (2013) 1:28–31. doi: 10.5694/mjao12.10628 25370281

