# Supplementary material for: Symptoms of anxiety and depression associated with steroid efficacy and clinical outcomes in patients with inflammatory bowel disease
Source: Front Psychiatry. 2023 Jul 21;14:1029467. doi: 10.3389/fpsyt.2023.1029467 (PMC10400767; doi:10.3389/fpsyt.2023.1029467)
Supplement: Supplementary file 1 [file Data_Sheet_1.docx]

Supplementary Materials

| 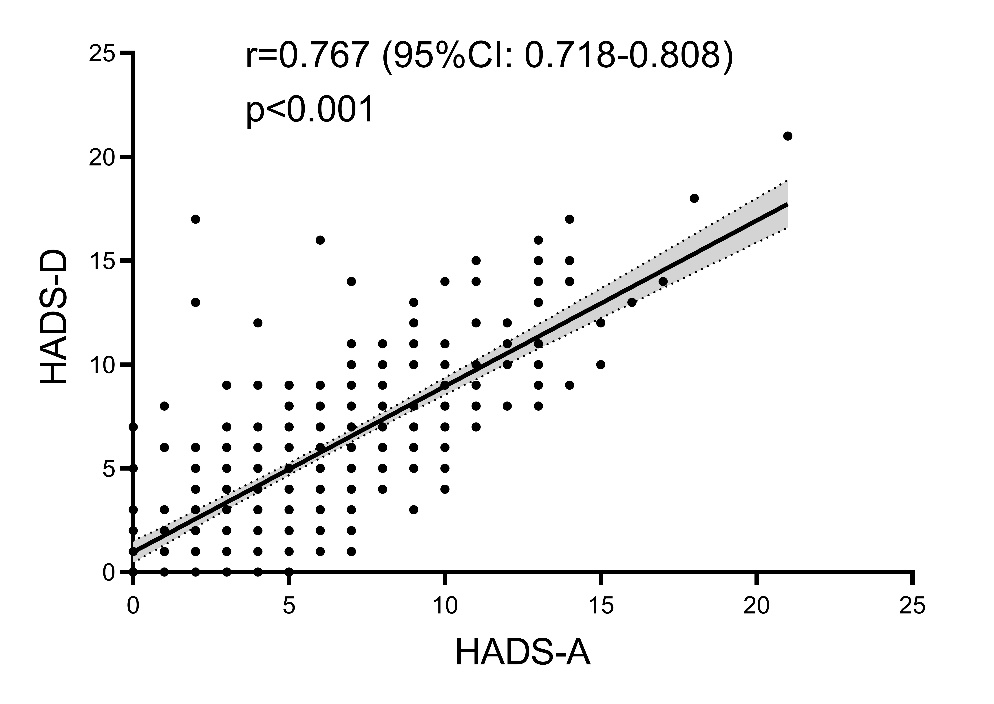  **Figure S1.** Correlation between the HADS-A and HADS-D score in IBD patients. HADS-A, the anxiety score of the Hospital Anxiety and Depression Scale; HADS-D, the depression score of the Hospital Anxiety and Depression Scale; r, correlation coefficient; CI, confidence interval.  **Table S1.** Multivariate logistic regression analysis of the association between anxiety/depression symptoms and therapy resistance. | | | |
| --- | --- | --- | --- |
| Variables | Model 1 | Model 2 | Model 3 |
| Steroids resistance  with anxiety/depression symptoms  AZA/6-MP resistance | 2.667 (1.215-5.852) | 2.725 (1.126-6.594) | 2.509 (1.012-6.220) |
| with anxiety/depression symptoms | 1.083 (0.375-3.130) | 1.246 (0.404-3.846) | 1.213 (0.368-3.997) |
| anti-TNF non-response |  |  |  |
| with anxiety/depression symptoms | 1.050 (0.434-2.539) | 0.940 (0.363-2.432) | 0.830 (0.285-2.417) |
| AZA/6-MP, azathioprine/6-mercaptopurine; Model 1: unadjusted; Model 2: adjusted for age, gender, BMI, tobacco use; Model 3: Model2+clinical active, endoscopically active, disease type, disease duration, and previous surgery. | | | |
